# Oncogenic Role of miRNA in Environmental Exposure to Plasticizers: A Systematic Review

**DOI:** 10.3390/jpm11060500

**Published:** 2021-06-02

**Authors:** Margherita Ferrante, Antonio Cristaldi, Gea Oliveri Conti

**Affiliations:** 1 Department of Medical, Surgical Sciences and Advanced Technologies “G.F. Ingrassia”, University of Catania, 95123 Catania, Italy; antonio.cristaldi@unict.it (A.C.); olivericonti@unict.it (G.O.C.); 2 Catania, Messina, Enna Cancer Registry, Via S. Sofia 87, 95123 Catania, Italy

**Keywords:** plasticizers, cancer, microRNA, in vitro study, PRISMA

## Abstract

The daily environmental exposure of humans to plasticizers may adversely affect human health, representing a global issue. The altered expression of microRNAs (miRNAs) plays an important pathogenic role in exposure to plasticizers. This systematic review summarizes recent findings showing the modified expression of miRNAs in cancer due to exposure to plasticizers. Following the Preferred Reporting Items for Systematic Reviews and Meta-Analyses (PRISMA) methodology, we performed a systematic review of the literature published in the past 10 years, focusing on the relationship between plasticizer exposure and the expression of miRNAs related to cancer. Starting with 535 records, 17 articles were included. The results support the hypothesis that exposure to plasticizers causes changes in or the deregulation of a number of oncogenic miRNAs and show that the interaction of plasticizers with several redundant miRNAs, such as let-7f, let-7g, miR-125b, miR-134, miR-146a, miR-22, miR-192, miR-222, miR-26a, miR-26b, miR-27b, miR-296, miR-324, miR-335, miR-122, miR-23b, miR-200, miR-29a, and miR-21, might induce deep alterations. These genotoxic and oncogenic responses can eventually lead to abnormal cell signaling pathways and metabolic changes that participate in many overlapping cellular processes, and the evaluation of miRNA-level changes can be a useful target for the toxicological assessment of environmental pollutants, including plastic additives and plasticizers.

## 1. Introduction

The continuous daily environmental exposure of humans to different chemicals may adversely affect human health, thus representing a global issue [1,2,3,4,5,6,7,8,9,10,11]. In the last decade especially, ecological and epidemiological studies have focused on the presence of plastics and their additives in food and the environment [6,12,13,14].

Plasticizers are added to plastics to increase their flexibility, durability, and pliability. A large broad of molecules are used by the polymer industry, including phthalates, bisphenolates, flame retardants, metals, parabens, polychlorinated biphenyls, tributyltin, organophosphate esters, etc.

Among the plasticizers, phthalates, are the most widely used in polyvinyl chloride (PVC), polyethylene terephthalate (PET), polyvinyl acetate (PVA), and polyethylene (PE). Phthalates can be found in toys, personal care products, food packages, paints, pharmaceuticals and drugs, medical devices, catheters, blood transfusion devices, cosmetics, and PVC products for home furnishing such as PVC films for floors or household accessories [12,15,16].

Bisphenol A (BPA), another plasticizer, is the major component in the manufacture of epoxy and polycarbonate plastics and flame retardants. The uses of BPA include coatings for PVC water pipe walls, plastic bottles for water, baby bottles, food packaging, receipt inks, cosmetics, plastic toys, etc. BPA has drawn attention from public health and governmental agencies due to its widespread use. Also, BPA exerts genotoxic and carcinogenic activities due to the similarity of its chemical structure that resembles that of diethylstilbestrol, an accredited human carcinogen [17,18]. Plasticizer exposure has been reported as a reproductive and developmental toxicant and carcinogen [19,20].

Plasticizers have been associated with hormone-sensitive cancers such as breast, prostate, endometrial, ovarian, testicular and thyroid cancers, but also with non-hormonally sensitive cancers such as cervical and lung cancers, osteosarcoma, and meningioma [21].

Based on the evidence that is already available, several countries regulated some plasticizers, especially in food and human consume products. Some laws and limits are formulated under which certain plasticizers are permitted to be used in the plastics industry, for instance, the USA Consumer Product Safety Improvement Act (CPSIA) [22], the Proposition 65 list of California State (PROP65) [23], the European Commission Regulation (EC) no. 372/2007 of 2 April 2007 [24], etc.

However, the Chang and Flow study [25] showed that also subchronic exposures to DEHP and DiNP in adulthood lead both to immediate and long-term reproductive consequences in female mice.

The altered expression of microRNAs (miRNAs) represents an epigenetic mechanism that exerts an important pathogenic role linked to exposure to environmental pollutants with several pathological outcomes, including cancer promotion and development [26,27,28]. These miRNAs are very-short RNA, ranging from 19 to 25 nucleotides in size, that regulate the post-transcriptional silencing of target genes. A single miRNA can target hundreds of mRNAs and influence the expression of a large number of genes often involved in several important functional pathways [26]. The miRNAs are differentially regulated in various types of cancer, including ovarian, liver, gastric, pancreatic, esophageal, colorectal, breast, and lung cancers [29].

An emerging hypothesis explores the supposed coordination between miRNA-mediated gene control and splicing events in gene regulatory networks [27]. Several studies suggest that the maturation of miRNAs may depend on splicing factors [30]. However, microRNA modification results in carcinogenesis only when other molecular changes occur simultaneously, such as suppression of the inhibition of the expression of mutated oncogenes, the formation of microRNA adducts, p53-microRNA interconnections, and alterations of the *Dicer* function [26].

Due to the considerable stability of miRNAs, they are measurable both in blood and tissues and are therefore eligible as potential biomarkers for several non-communicable diseases, including cancer [29].

This systematic review summarizes recent findings showing the aberrant expression of miRNAs in cancer due to plasticizer exposure. We further discuss the challenges in environmental-miRNA research because this approach can be key for understanding the mechanism of cancer pathophysiology but also for early screening and/or personalized cancer therapy.

## 2. Materials and Methods

A brief critical review of scientific papers from the last ten years selected using the PubMed, Scopus, and Web of Science databases was carried out. The “Preferred Reporting Items for Systematic Reviews and Meta-Analysis” (PRISMA) methodology was applied in this study.

To assess the influence of plasticizer exposure on the expression of miRNAs in humans, all original articles published from 1 January 2010 to 29 December 2020 were selected based on the following criteria:

- the articles are original,
- articles report on plasticizer or plasticizer exposure,
- articles report on miRNAs analysis and identification,
- articles have correct scientific methodology,
- articles include the identification of miRNAs for cancers of all target organs.

We searched the databases for controlled randomized studies, cohort studies, case–control studies, case reports, and in vitro studies. Only original articles written in English were collected for the PRISMA review.

We excluded papers that did not include original data such as informative reviews, commentaries, and editorials. Systematic reviews and meta-analyses were not eligible, but their references were screened for recovery of eligible studies missing in the databases.

Two investigators (A.C. and G.O.C.) screened all citations for potentially eligible studies and extracted data independently. Disagreements were adjudicated by a third investigator (M.F.).

The research was conducted using keywords including “Plasticizers and microRNA”, “Plasticizers and oncogenic microRNA”, “additives of plastic and miRNA cancer“, “endocrine disrupting chemicals and miRNA cancer”, “BPA and miRNA cancer”, “di-n-butyl phthalate and miRNA cancer”, “DBP and miRNA cancer”, “monobutyl phthalate and miRNA cancer”, “MBP and miRNA cancer”, “Organophoshorus flame retardants and miRNA cancer”, “flame retardants and miRNA cancer”, “di(2-ethylhexyl) phthalate and miRNA cancer”, “DEHP and miRNA cancer”, “mono-(2-ethylhexyl) phthalate and miRNA cancer”, “MEHP and miRNA cancer”, “methylparaben and miRNA cancer”, “parabens and miRNA cancer”, “phthalates and miRNA cancer”, “environmental phenols and miRNA cancer”, “organophosphate esters and miRNA cancer”, “Tributyltin and miRNA cancer”, “PCBs and microRNAs cancer”, “butylbenzyl phthalate and miRNA cancer”, “PVC and miRNA cancer”.

We also used combinations of the keywords, such as “Plasticizers and oncogenic effects” and “Plasticizers and microRNA changes”.

All eligible studies were evaluated as eligible using a modified Newcastle Ottawa scale (rating system to score each study) [4].

## 3. Results

### 3.1. Literature Inclusion Criteria

**Figure 1:**
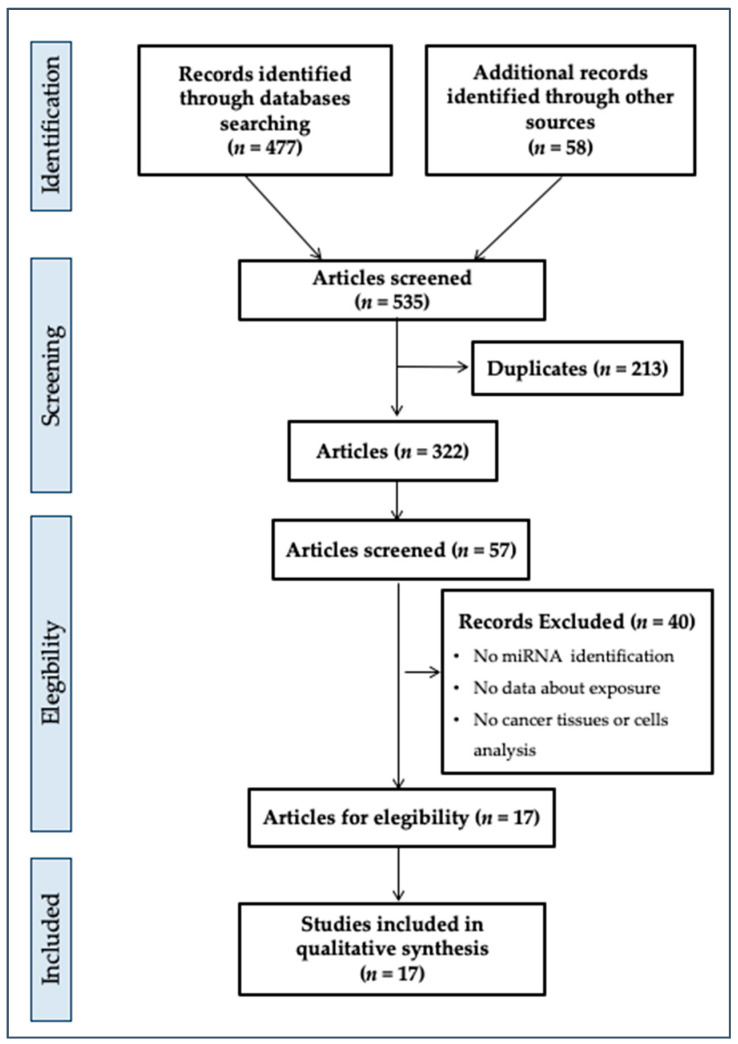
PRISMA flow diagram.

Our initial search produced 477 potential references (Figure 1) from databases and 58 references from other sources.

Starting with 535 records, we identified in the first phase 322 records after the deletion of 213 duplicates. In addition, via evaluation of the title and abstract, we screened 57 records. Of these, 40 records were excluded due to the absence of some criteria of eligibility (reporting on miRNAs or indications, evidence about exposure to plasticizers, cancer tissue or cells analysis reporting, correct sample size, in vivo or in vitro study, statistical analysis of data, analysis of plasticizers in biological tissues) as listed in Figure 1. Finally, we included 17 studies in the systematic review. These studies used various approaches or study designs, but all focused on the effects of exposure to plasticizers on outcomes defined as “oncogenic miRNA identification and their down- or upregulation description”.

### 3.2. Summary of Literature Included

Figure 1 describes the findings of the performed collection and screening procedures.

We included the following studies, in chronological order: Tilghman et al., 2012 [31], Meng et al., 2013 [32], Li et al., 2014 [33], Kim et al., 2015 [34], Buñay et al., 2017 [35], Chang et al., 2017 [36], Chou et al., 2017 [37], Hui et al., 2018 [38], Wu et al., 2018 [39], Yin et al., 2018 [40], Scarano et al., 2019 [41]; Wang et al., 2019 [42], Zhu et al., 2019 [43], Cui et al., 2019 [44], Chorley et al., 2020 [45]; Duan et al., 2020 [46], and Zota et al., 2020 [47].

All the studies are in vitro cell studies on rats, mice, or human cancer cell lines. Several plasticizers were studied through controlled in vitro exposure as reported in Table 1.

Human endometrial, hemangioma, acute myeloid leukemia, ovarian, breast, hepatocellular, oral squamous, and prostate cancer cells lines were evaluated.

### 3.3. Detailed Overview of the Literature Included

#### 3.3.1. In Vitro Studies

Tilghman et al. (2012) [31] studied the effects of BPA (10 μM) and DDT (10 μM) on miRNA regulation and expression levels in hormone-responsive human breast cancer cells. The MCF-7 breast cancer cell line showed that both pollutants increased the expression of *ER* receptor target genes, including the progesterone receptor, bcl-2, and trefoil factor. The revealed miRNAs (27) were outlined in the exposed cells (miR-21, miR-638, miR-663, miR-1915, let-7g, let-7c, miR-923, miR-93, miR-320a, miR-1308, let-7f, miR-15b, miR-1275, miR-27b, miR-222, miR-193a-5p, miR-16, miR-26b, miR-149, miR-92a, miR-99b, miR-92b, miR-342-3p), of which several were upregulated and downregulated according to Table 1.

Several genes were differentially regulated by the compounds. For example, *Jun* and *Fas* genes were increased approximately 1.8- and 1.5-fold by BPA, but were relatively unchanged by DDT. The onco-miR-21 is an estrogen-regulated miRNA that plays an important role in breast cancer. In this study, miR-21 expression was downregulated by BPA, and several members of the let-7 family (let-7a, let-7b, let-7c, let-7d, let-7e and let-7f), were downregulated (*p* < 0.05) by all treatments. In contrast, miR-638 (*p* < 0.005), miR-663 (*p* < 0.005), and miR-1915 (*p* < 0.005) were upregulated by BPA and DDT.

Chou et al. (2017) [37] investigated the role of BPA exposure in the disruption of miRNA regulation and whether the related gene expression is decisive for carcinogenic progression. This study was carried out using human endometrial cancer RL95-2 cells and treatment with low to moderate BPA concentrations (10, 10^3^, and 10^5^ nM).

Chou and colleagues reported that BPA exposure reduced miR-149 expression, downregulating the DNA repair gene *ARF6* (ADP ribosylation factor 6) and tumor protein p53 (*TP53*) and upregulating *CCNE2* (cyclin E2). The results of the study also showed that BPA was able to increase miR-107 to suppress hedgehog signaling factors, acting as a suppressor of fused homologs (*SUFU*) and GLI family zinc finger 3 (*GLI3*) and providing proof of the potential epigenetic mechanism of BPA exposure on endometrial carcinogenesis risk. In fact, miR107, miR149, miR200c, miR203, miR205, and miR765 changed the expression of some genes (*TP53*, *JUN*, *LAMB4*, *CCCDC6*, *PRKCA*, *STAT1*, *SUFU*, *CXCL8*, *DVL1*, *GLI3*, *CRK*, *LAMC1*, *MAPK1*, *MAPK9*) involved in the cancer pathway, recording a significant fold change of *N* > 2.0 compared to the control.

This study permitted the discovery and identification of five relevant pathways for potential BPA-induced endometrial cancer progression, including the cancer pathway, hedgehog pathway, cell cycle, adherens junction, and MAPK signaling pathway. In addition, *TP53*, *GLI3*, *CCNE2*, *CRK*, *KIF23*, *SAMD2*, *CCDC6*, *FZD3*, *ARF6*, *MAPK9*, *SUFU*, *PRC1*, *MDM2*, *SMAD4*, *DVL1*, *EGLN1*, *JUN*, *MYC*, *LAMC1*, *PRKACA*, and *STAT1* were genes that overlapped and were expressed significantly differently in these five pathways.

Meng et al. (2013) [32] developed an miRNA biosensor and applied this novel tool to detect miRNA-21 extracted from human hepatocarcinoma BEL-7402 cells and human mastocarcinoma MCF-7 cells and their expression under in vitro exposure to BPA. Normal human hepatic L-02 cells, BEL-7402 cells, and MCF-7 cells were incubated with 100 μM BPA at the same concentration for three and five days, respectively. The expression profiles of miRNA-21 in BEL-7402 and MCF-7 became 1.415-fold and 1.468-fold higher than that of normal L-02 cells, respectively, showing that the miRNA expression levels of cancer cells were upregulated compared to normal cells.

Li et al. (2014) [33] studied how microRNAs are involved in curcumin-mediated protection from BPA-associated induced effects on a breast cancer MCF-7 cell line. The MCF-7 cell line was exposed to BPA for 4 days. The results showed that BPA exhibited estrogenic activity by increasing the proliferation of estrogen-receptor-positive MCF-7 human breast cancer cells and promoting the transition of the cells from the G1 to S phase. Curcumin was able to inhibit the proliferative effects of BPA on MCF-7 cells. In addition, the BPA-induced upregulation of oncogenic miR-19a and miR-19b and the dysregulated expression of miR-19-related downstream proteins, including *PTEN*, *p-AKT*, *p-MDM2*, *p53*, and proliferating cell nuclear antigen, were sufficiently reversed by curcumin. Furthermore, Li and colleagues highlighted the important role of miR-19 in BPA-mediated MCF-7 cell proliferation, suggesting for the first time that curcumin modulates the miR-19/*PTEN*/*AKT/p53* axis to exhibit its protective effects against BPA-associated breast cancer.

Kim et al. (2015) [34] used HepG2 cells that are widely used as a model system for studies of liver metabolism and genotoxicity. In particular, the authors determined the role of BPA exposure in the epigenetically affected expression of miR-22. The authors found methylated Chr17:1565786-1565940 regions (promoter sites for miR-22) in the normal samples, but unmethylated ones in samples exposed to BPA. Kim et al. identified seven differentially expressed miRNAs, including miR-22, in the BPA-exposed sample vs. the control. Notably, in samples exposed to BPA, miR-22 showed a 3.38-fold upregulation compared to normal samples. The study results highlight the regulation of miR-22 expression via hypomethylation of the promoter region due to BPA exposure.

Hui et al. (2018) [38] focused on BPA and ovarian cancer. This study was performed using in vitro exposure to BPA (10 or 100 nM) or 0.1% DMSO for 24 h using human ovarian adenocarcinoma SKOV3 cells, and then, the global gene expression profile was determined via high-throughput RNA sequencing. Transcriptomic analysis revealed 94 different expression genes related to tumorigenesis and metastasis.

The authors revealed the upregulation of miR-21-5p and miR-222-3p, also reporting that BPA (10 and 100 nM) increased migration and invasion as well as induced epithelial to mesenchymal transitions in SKOV3 and A2780 cells. Accordingly, doses of BPA found in the environment are capable of activating the regular Wnt signaling pathway. This study analyzed the possible mechanisms underlying the effects of BPA on ovarian cancer. Environmentally relevant doses of BPA modulated the gene expression profile and promoted the progress of epithelial to mesenchymal transition via the canonical Wnt signaling pathway of ovarian cancer.

Wu et al. (2018) [39] showed that BBP induced the proliferation of both ER(+) MCF-7 and ER(−) MDA-MB-231 breast cancer cells. This was proven by the increased cell viability, the transition of the cell cycle from the G1 to the S phase, the upregulation of *PCNA* and *Cyclin D1*, and the downregulation of *p21*. Moreover, BBP modulated the expression of the oncogenic miR-19a/b and *PTEN/AKT/p21* axis, revealing that miR-19 plays a crucial role in the promoting effect of BBP on breast cancer cells via the targeting of PTEN 3’UTR. These findings provide an important tool for targeted cancer intervention.

Zhu et al. (2019) [43] investigated the role of BBP in the cell proliferation of prostate cancer cells. Human prostate cancer LNCaP and PC-3 cell lines were exposed to low doses (0, 10^−4^, 10^−5^, 10^−6^, 10^−7^ and 10^−8^ mol/L) of BBP for 6 days. Zhu’s results showed that 10^−6^ and 10^−7^ mol/L BBP increased the expression of *cyclinD1* and *PCNA*, decreased *p21* expression, and induced cell growth in both LNCaP and PC-3 cells vs. the control group. Furthermore, the authors found that BBP significantly downregulated the expression of miR-34a, along with upregulating miR-34a target gene *c-Myc*. Via cell transfection of an miR-34a mimic and inhibitor, the authors demonstrated that, in prostate cancer cells, the BBP trigger promoted cell proliferation mediated through the miR-34a/c-myc axis.

Duan et al. (2020) [45] also studied the effects of BBP on human acute monocytic leukemia AML U937 (isolated from the histiocytic lymph), Raji (lymphoblast Burkitt’s lymph), and HL-60 (a promyelocytic cell line) cell lines, and normal blood cells.

BBP doses of 10^−9^ and 10^−4^ M were used for investigating the potential effect of BBP on the malignancy of AML cells. Instead for carrying out a mechanistic study, a dose of 10^−8^ M was used. The authors examined the effects of BBP on the proliferation of AMLU937, Raji, and HL-60 cell lines. Moreover, the authors verified BBP’s perturbation of treated U937 cells against the efficacy of chemotherapy using a double-exposure with increasing concentrations of daunorubicin or cytarabine with or without 10^−8^ M BBP.

The results revealed that (10^−8^ M) BBP can induce the proliferation and reduce the chemotherapy sensitivity of acute monocytic leukemia cells. *PDK1*, *PDK2*, *PDK3*, *PDK4*, *PDP2*, and *PDPR* genes can regulate the glucose metabolism and glycolysis of cancer cells. In fact, cancer cells are characterized by high rates of glycolysis. The pyruvate dehydrogenase kinase (*PDK*) supports these energetic needs and also favors apoptosis resistance. Duan showed that BBP increased the expression of *PDK4* and *PDP2* in U937 cells, while in Raji cells, BBP only increased the expression of *PDK4*. This study confirmed that BBP can decrease the expression of miR-15b-5p, while it had no effect on miR-182 in both U937 and Raji cells. The overexpression of miR-15b-5p can abolish the BBP-induced mRNA and protein expression of *PDK4* in U937 cells. Furthermore, the inhibitor of miR-15b-5p can increase the mRNA and protein expression of *PDK4* in U937 cells.

Duan’s results suggested that the downregulation of miR-15b-5p was involved in BBP-induced *PDK* and demonstrated that BBP can increase the mRNA stability of *PDK4* via the downregulation of miR-15b-5p.

Hence, BBP had no effect on the transcription and protein stability of *PDK4*; however, it significantly increased the mRNA stability of *PDK4.*

In Yin et al. (2018) [41], the global alterations of miRNA and mRNA expression in juvenile rat Sertoli cells (SCs) treated with 0.1 mM MBP were evaluated. Yin’s results revealed that miR-3584-5p and miR-301b-3p were upregulated and their common target gene, Dexamethasone-induced Ras-related protein 1 (*Rasd1*), was downregulated. SC proliferation induced by low MBP concentration in vitro could be mediated by *Rasd1* regulation of the *ERK1/2* signaling pathway. These results represent a possible avenue to apply personalized medicine screening and therapy in testicular tumors induced by exogenous chemicals.

Cui et al. (2019) [44] studied the potential influence of MEHP, DEHP, DCHP, and BBP on the progression of hemangioma, one of the most common tumors of infancy. This in vitro study was carried out using hemangioma cells. The authors found that 100 nM of BBP can significantly trigger the migration and invasion of hemangioma cells, also inducing the overexpression of *Zeb1,* a powerful transcription factor for cell migration and invasion, via miR-655 suppression or downregulation. As 100 nM of BBP might also be found in human tissues, the potential health risks of BBP, particularly for oncologic HA patients, should be given more attention.

#### 3.3.2. In Vivo Studies

Buñay et al. (2017) [35] studied the consequences of chronic exposure to a mixture of phthalates and alkylphenols for the testes of male mice and in particular, reported changes in the expression patterns of miRNA/isomiRs, which act as regulators of gene expression in the testes. Additionally, damage to the testis and changes in the genes responsible for encoding proteins involved in the biogenesis, processing, editing, stability, or degradation of miRNAs were assessed. Buñay et al. carried out a case-control exposure study on a mix of phthalates and alkylphenols using adult male mice.

The exposed mice showed the degeneration of seminiferous tubules and hypertrophy/hyperplasia in Leydig cells and also an increase in exfoliation of germ cells of seminiferous tubules that close the lumen or showed fully closed tubules. Regarding mRNA levels, the authors report that the miRNAs of *Star* and *Cyp17a1* and *Sp1* and *Cyp11a1* were upregulated and downregulated, respectively. Instead, no significant differences in *Hsd3b1* mRNA expression were detected.

The authors quantified the mRNA expression levels of genes encoding proteins that are involved in pri-miRNA processing (*Drosha*), nuclear export (*Xpo5*), stability/degradation (*Lin28*, *Zcchc11*, *Zcchc6*, and *Snd1*), editing (*Adar*) and processing of pre-miRNAs (*Dicer*, *Ago2*).

A significant increase in the mRNA levels of *Drosha*, *Adar*, and *Zcchc11* in the testes of exposed mice was found compared to control mice, contrary to *Zcchc6*, *Dicer*, *Xpo5*, *Ago2*, *Lin28b,* and *Snd1*, which showed no differences.

miR20b-5p and miR-1291, which are implicated in cancer, and miR-3085-3p, implicated in inflammation, were all downregulated. miR-1291 targets DNA methyltransferases (*Dnmt3a*, *Dnmt3b*) that are involved in (de novo) histone methylation, genomic imprinting, X-chromosome inactivation, and testicular germ cell tumors due to exposure to alkylphenols. In addition, *Ccnd2*, *Ccnd1*, and *Raf1* are targets of the downregulated miR-15b-5p in exposed mice, and these targets are implicated in cancer and cell cycle regulation. Hence, this study suggests that the downregulation of sncRNAs through miR-1291 related to exposure to plasticizer mixtures might promote changes in the DNA methylation pattern, causing the epigenetic transmission of several diseases, including cancer.

In Chang et al. (2017) [36], the role of MEHP-induced reactive oxygen species (ROS) for genotoxicity was explained. Mono-ethylhexyl phthalate (MEHP) is a metabolite of DEPH. The toxicity of MEHP is more potent than that of DEPH. Chang’s study provided evidence of the carcinogenicity of MEHP in Chinese hamster AA8, UV5, and EM9 ovary cells, as well as its ability to induce epigenetic modifications.

The cell lines were exposed to 0, 10, 25, and 50 mM MEHP. However, at 50 mM MEHP, all the cells died. The protection was not significant at 25 mM MEHP, and, even after exposure to a lower dose of MEHP (1 mM), the PARP-1-KD cells had a higher level of single-strand breaks. The subsequent *gpt* gene sequencing used to analyze the mutation points on the genes of AS52 mutant cells (ASMC) showed that 90% of all mutations were single-base pair substitutions, especially G:C to A:T mutations. Independent AS52-mutant cell clones were collected and used to perform sequential in vivo xenograft tumorigenic studies, and 4 of 20 clones had aggressive tumor growth. The study also showed that miR-let-7a and miR-125b has been downregulated in ASMC, which might raise oncogenic MYC and RAS levels and promote the activation of the *ErbB* pathway. The mutagenic pathway of MEHP can probably be triggered via the generation of ROS, causing base excision damage and resulting in carcinogenicity.

Wang et al. (2019) [48] sought to evaluate the capability of MEHP to promote the proliferation of oral cancer through an in vitro/in vivo study using human oral squamous carcinoma (OSCC) (human OSCC SCC-4, SCC-9, and SCC-25) cells and cell nuclear antigen (PCNA). SCC-4 cancer cells (2 × 106 per mouse) were diluted in 100 μL of normal medium and a researcher injected these subcutaneously into the left flank of each mouse to obtain OSCC cancer xenografts. When the tumor grew to 100 mm^3^, the mice of the MEHPs group were treated with MEHP (4 mg per kg, body weight) via intratumoral injection four times every three days. Tumor volume was measured every three days and, at the end of the experiment, mice were sacrificed and the xenograft tumors were removed to measure the expression of miRNAs and proteins.

The authors supported their hypothesis with results that showed the proliferation of oral cancer via MEHP through the downregulation of miR-27b-5p and miR-372-5p. In addition, MEHP induced the expression of *c-Myc*, which can suppress the transcription of miR-27b-5p in OSCC cells. Therefore, Wang’s study showed that MEHP can promote the growth and progression of OSCC via the downregulation of miR-27b-5p and miR-372-5p.

Scarano et al. (2019) [41] studied the genome-wide levels of mRNAs to determine if perinatal exposure to a phthalate mixture in pregnant rats was capable of modifying gene expression during the prostate development of the filial generation. The study sought to determine the epigenetic role of these pollutants in prostate cancer.

Pregnant female Sprague Dawley rats were exposed daily (from gestational day 10 to postnatal day 21) to a mixture of phthalate by gavage and were suppressed after. Four groups were established—a control group exposed only to corn oil; (T1) 20 mg of the mixture (20 mg/kg/day); (T2) 200 mg of the mixture (200 mg/kg/day); and (T3) 200 mg of the mixture (200 mg/kg/day). The cocktail contained DEHP, DEP, DBP, DiBP, BBzP, and DiNP. The two lower doses mimicked daily human exposure levels based on the amount of DEHP, and the higher dose was selected to compare our results with those of similar phthalate studies. Rats from groups T1 to T3 received the respective doses of the phthalate cocktail prepared with 21% DEHP, 35% DEP, 15% DBP, 8% DiBP, 5% BBzP, and 15% DiNP. After birth, the number of F1 offspring per litter was reduced to 8 (at a 1:1 ratio between males and females whenever possible), and litters with fewer than six pups were suppressed.

miRNAs in the treated groups versus the control were upregulated in T1 vs. C and in T2 vs. C. miR-141-3p was exclusively upregulated in the T1 vs. C group, whereas other miRNAs, such as miR-30d-5p, were deregulated in both groups with weak but significant alterations in gene expression. miRNA-184 was upregulated in all treatment groups vs. C. Among the possible targets for miR-141-3p (53 targets), 51 were downregulated. The MiRNAs differentially expressed in the prostate tissue of these exposed animals were elicited in Table 1. Scarano’s study, based on the evaluation of miRNAs and histopathological and immunostaining analyses, support the hypothesis of the epigenetic role of phthalate in prostate oncogenesis.

Chorley et al. (2020) [46] measured liver and blood miRNAs in male B6C3F1 mice exposed both to a known chemical activator of the peroxisome proliferator-activated receptor alpha (PPARα) and DEHP, respectively, for 7 and 28 days at concentrations of 0, 750, 1500, 3000, and 6000 ppm through oral exposure (feed). The PPARα pathway is a common target of several environmental chemicals. At the highest DEHP dose tested, 61 miRNAs were altered after 7 days, and 171 miRNAs after 28 days of exposure, with 48 overlapping miRNAs. Analysis of the 48 common miRNAs indicated the enrichment in PPARα–related targets and other pathways related to liver injury and cancer. The experiment was repeated using mmu-miRs-182-5p and -378a-3p analysis for DEHP, as well as di-n-octyl phthalate (DNOP) and n-butyl benzyl phthalate (BBP), two other related phthalates with weaker PPARα activity.

The results showed that the deregulatory potency of DEHP was superior to DNOP and BBP, and mmu-miRs-125a-5p, -182-5p, -20a-5p, and -378a-3p showed a clear dose relation linked to the PPARα pathway. These findings also highlight the putative miRNA biomarkers, as well as the stratified chemical potency of plasticizers and environmental pollutants in general.

Zota et al. (2020) [47] conducted the only human study included in this review. The Fibroids Observational Research on Genes and the Environment (FORGE) study involved 45 women living in Washington, DC, from 2014–2017. Eligible women were nonpregnant, pre-menopausal, English-speaking, and ≥18 years of age. The authors quantified the expression levels of 754 miRNAs in fibroid tumor samples and analyzed spot urine samples for phthalate metabolites collected from women undergoing surgery for fibroid treatment.

Associations between the miRNA levels in fibroids and phthalate biomarkers were also evaluated using a linear regression adjusted for age, race/ethnicity, and body mass index (BMI), and all the statistical tests were adjusted for multiple comparisons.

Fibroid tissues were collected during hysterectomy or myomectomy procedures. For patients with multiple fibroids, only the largest fibroid was sampled.

In addition, the evaluation of single metabolites was carried out, including diethyl phthalate (DEP), monoethyl phthalate (MEP), di-n-butyl phthalate (DnBP), mono-n-butyl phthalate (MnBP), mono-hydroxybutyl phthalate (MHBP), diisobutyl phthalate (DiBP), monoisobutyl phthalate (MiBP), mono-hydroxyisobutyl phthalate (MHiBP), butylbenzyl phthalate (BBzP), monobenzyl phthalate (MBzP), DnOP, mono(3-carboxypropyl) phthalate (MCPP), diisononyl phthalate (DiNP), monocarboxyoctyl phthalate (MCOP), diisodecyl phthalate (DiDP), monocarboxynonyl phthalate (MCNP), di(2-ethylhexyl) phthalate (DEHP), mono(2-ethylhexyl) phthalate (MEHP), mono(2-ethyl-5-hydroxyhexyl) phthalate (MEHHP), mono(2-ethyl-5-oxohexyl) phthalate (MEOHP), and mono(2-ethyl-5-carboxypentyl) phthalate (MECPP). The authors also calculated two summary measures, the molar sum of DEHP metabolites (ΣDEHP)21 and a potency-weighted sum of antiandrogenic phthalate metabolites (ΣAA phthalates).

The fibroid characteristics were similar across racial/ethnic groups. Phthalate exposure was ubiquitous in the enrolled woman, but nine phthalate metabolites were detected in >90% of participants. However, MEP levels were significantly higher in Black women. The enrolled women were Black (62%), overweight or obese (76%), privately insured (64%), and undergoing a myomectomy (58%). Compared with White/Latina women, Black women were more likely to be obese, publicly insured, and undergoing hysterectomy. The miRNA profiles detected were surprising with respect to social determinants.

A total of 35 miRNAs were underexpressed, and 39 miRNAs were overexpressed in fibroids rather than myometrium. Also, the expression of miR-10a-5p, miR-10a-3p, miR-140-3p, miR-144-5p, miR-150-5p, miR-205-5p, miR-27a-5p, miR-29b-2-5p, miR-29c-5p, miR-451a, and miR-95-3p was three-fold greater in myometrium; while expressions of miR-135a-5p, miR-135b-5p, miR-137-3p, miR-302b-3p, miR-335-3p, miR-34a-5p, miR-34a-3p, miR-34b-5p, miR-34c-5p, miR-483-5p, miR-488-3p, miR-488-5p, miR-508-3p, miR-577, miR-592, miR-651-5p, miR-885-5p, and miR-9-3pthese miRNAs were three-fold greater in fibroids.

The authors found 285 significant associations between phthalate biomarkers and miRNAs (*p* < 0.05), 34 of which were significant at *p* < 0.005.

After adjusting for multiple testing, we found two miRNAs associated with phthalate biomarkers—MHBP, associated with an increase in miR-10a-5p of 0.76 (95% CI = (0.40, 1.11)), and MEHHP, associated with miR-577 (β = 1.06, 95% CI = (0.53, 1.59)). Eight phthalate-miRNA associations varied significantly between White/Latina and Black women, and among these, there was an association between MBzP and miR-494-3p. Also, among white/Latina women, there were associations between MCPP and miR-337-5p; MBzP and miR-1227-3p; MEP and miR-645; MEP and miR-564; MEP and miR-374-5p; MEHP and miR-128-3p; and MEHP and miR-337-3p. Ten miRNAs were significantly associated with phthalate biomarkers either in the main analysis or in racial groups (miR-10a-5p, miR-577 miR-494-3p, miR-337-5p, miR-1227-3p, miR-645, miR-564, miR-374a-5p, miR-128-3p, miR-337-3p).

Zota et al. identified 923 mRNA targets that were experimentally observed or highly predicted targets of the 10 miRNAs, but 3 miRNAs (miR-10a-5p, miR-128-3p, miR-494-3p) were significantly associated with multiple fibroid-related processes, including angiogenesis, apoptosis, the proliferation of connective tissues, cell viability, tumorigenesis of the reproductive tract, and smooth muscle tumors.

miR-10a, miR-150, miR-29b, miR-29c, and miR-451 were underexpressed, and miR-34a was overexpressed in fibroids. The authors reported that miR-10a-5p expression in particular is associated with concentrations of MHBP, an oxidative metabolite of DnBP which is found in some personal care products, demonstrating that the epigenome is sensitive to interactions between chemical and non-chemical stressors, but also to social determinants that can influence a wide range of physical and social environmental exposures altering the biological response to environmental pollutants. On the basis of these results, the lack of human studies needs to be addressed urgently.

## 4. Discussion and Conclusions

The epigenetic effects of environmental chemicals such as plasticizers, including BPA and phthalates, on DNA methylation, as well as the expression of miRNAs, have substantiated our knowledge about the etiology of chronic diseases in humans, such as cancer. Evidence from in vitro and in vivo models has proved that epigenetic modifications due to exposure to common environmental pollutants can induce alterations in gene expression that may persist throughout life, increasing susceptibility to cancer. Epigenetics can affect the gene expression profiles of various organs and tissues. Among the phthalates, BPA, DEHP, MEHP, DBP, BBP, and MBP were found to cause 1232 and 265 interactions with the same genes and proteins, respectively.

This systematic review shows that miRNA-based diagnostic models can predict several targets of cancerous organs targets in humans with high accuracy. Also, the evidence regarding the carcinogenicity of several plasticizers was further supported by expression studies, permitting the future use of specific miRNA as valuable predictor or screening method for early diagnosis biomarkers as showed by Meng et al. study [32].

The use of profiling of miRNA as screening test through the high-throughput omic methods (microarrays and real-time quantitative PCR or qPCR, as well as real time PCR and next-generation sequencing) should be improved and applied in molecular early diagnosis to identify novel oncogenes, mechanisms, and/or pathways in which a stimuli, whether genetic or environmental, exerts a change on cell physiology to an oncological status.

Although the use of miRNAs is currently applied as a basic science tool, the overall miRNA’s gene expression is moving from research laboratories to the large-scale clinical trials for the validation of a new diagnostic tool or for allowing clinical states to be determined in diseases such as cancer or other miRNA-diseases or altered gene expression related diseases. The use of miRNAs, as non-invasive tool of early diagnosis, need to be implemented in the clinical approach and miRNAs may be promising and effective candidates in the development of highly sensitive, noninvasive biomarkers for tumors screening prevention.

The miRNA-level changes can be useful for the toxicological assessment of several environmental pollutants, including plastic additives and plasticizers.

In this review, we showed that the interaction of plasticizers with several redundant miRNAs such as let-7f, let-7g, miR-125b, miR-134, miR-146a, miR-22, miR-192, miR-222, miR-26a, miR-26b, miR-27b, miR-296, miR-324, miR-335, miR-122, miR-23b, miR-200, miR-29a, and miR-21 might induce deep alterations in miRNA-mediated regulation and functions. These genotoxic and oncogenic responses can eventually lead to abnormal cell signaling pathways and metabolisms that participate in many intercrossed or overlapped cellular processes.

BPA induces the hypomethylation of histone promoter regions, indicating methylation changes as one of the possible mechanisms of BPA-induced adverse effects on carcinogenesis. BPA is also involved in the downregulation of gene repair ARF6 (involved in cell differentiation, apoptosis, and cell regulation), *TP53* (a tumor suppressor gene also referred to as the “Guardian of the Genome”), and over-regulates *CCNE2,* which is able to interact with *CDKN1A* and *CDKN1B* proteins, and with *CDK3*. The aberrant expression of *CCNE2* is a cause of cancer [48].

Phthalates downregulated the activity of some miRNAs (see Tab.1) implicated in cell cycle regulation and cancer. Additionally, the activation and overexpression of *ErbB*, *PPARα* pathways, the generation of ROS, and the overexpression of *Zeb1* (a transcription factor involved in cell migration and invasion) resulted from phthalate exposure.

It is important to note that the machinery by which plasticizers alter the epigenetic assets of cells require further study to elucidate the biology and biochemistry relatively to epigenetic alterations but also to disease-associated epigenetic alterations. A better understanding of these mechanisms will lead to better prediction of the health effects of plasticizers, allowing more targeted, easy, and appropriate disease-prevention and therapy strategies [49,50].

The lack of human studies needs to be addressed. Experimental evidence will permit the proposal of dedicated epidemiological studies to evaluate the real effects of plasticizers on human health, especially for cancer derived by microplastics and their plasticizers that are yet to be properly studied by oncologists yet.

## Figures and Tables

**Table 1:** The included studies and their results ^++^.

Study	In Vitro/Vivo	Plasticizer	miRNA	Expression	Reference No.
Wu et al., 2018	^^^ MCF-7 ^^^ MDA-MB-231	BBP	miR-19a	Up	[39]
			miR-19b	Up	
Zhu et al., 2019	^π^ LNCaP and PC-3cells	BBP	miR-34a	Down	[43]
Duan et al., 2020	^β^ AML U937, Raji, and HL-60 cell lines.	BBP	miR-15b-5p	Down	[45]
			miR-182	NE	
Chou et al., 2017	** RL95–2 cell line	BPA	miR-107	Up	[37]
			miR-203	Up	
			miR-205	Up	
			miR-103a	Up	
			miR-200c	Up	
			miR-141	Up	
			miR-221	Up	
			Let-7a-5p	Up	
			miR-193b	Up	
			miR-423	Up	
			miR-513	Down	
			miR-149	Down	
			miR-765	Down	
Tilghman et al., 2012	^^^ MCF-7 cell line	BPA	miR-21	Down	[31]
			let-7g	Down	
			let-7c	Down	
			miR-923	Down	
			let-7f	Down	
			miR-15b	Down	
			miR-27b	Down	
			miR-26b	Down	
			miR-342-3p	Down	
			miR-638	Up	
			miR-663	Up	
			miR-1915	Up	
			miR-93	Up	
			miR-320a	Up	
			miR-1308	Up	
			miR-1275	Up	
			miR-222	Up	
			miR-149	Up	
	^ρ^ MCF-7F cells		miR-21	Up	
Meng et al., 2013	^$^ BEL-7402 cells	BPA	miR-21	Down ^a^/Up ^b^	[32]
	^^^ MCF-7 cells		miR-21	Down ^a^/Up ^b^	
Li et al., 2014	^^^ MCF-7 cell line	BPA	miR-19°	Up	[33]
			miR-19b	Up	
Kim et al., 2015	° HepG2 cell line	BPA	miR-22	Up	[34]
Chou et al., 2017	** RL95–2cell line	BPA	miR-107	Up	[37]
			miR-203	Up	
			miR-205	Up	
			miR-103a	Up	
			miR-200c	Up	
			miR-141	Up	
			miR-221	Up	
			Let-7a-5p	Up	
			miR-193b	Up	
			miR-423	Up	
			miR-513	Down	
			miR-149	Down	
			miR-765	Down	
Hui et al., 2018	^§^ SKOV3 and ^§^ A2780 cell lines	BPA	miR-21-5p miR-222-3p	Up Up	[38]
Yin et al., 2018	Juvenile rat Sertoli cells	MBP	miR-199a-3p	Up	[40]
			miR-301b-3p	Up	
			miR-3584-5p	Up	
Chang et al., 2017	* AS52 CHO cells inoculated in mouse	MEHP	miR-let-7a	Down	[36]
			miR-125b-5p	Down	
			mir-130a-3p	Down	
			miR-27a-3p	Down	
			miR-25-3p	Down	
			miR-92a-3p	Down	
Wang et al., 2019	In vivo ^Σ^ OSCC cells/subcutaneously injected in mice	MEHP	miR-27b-5p	Down	[48]
			miR-372-5p	Down	
Buñay et al., 2017	In vivo Adult mice	Cocktail (DEHP, DBP, BBP, NP, OP)	miR20b-5p	Down	[35]
			miR-1291	Down	
Cui et al., 2019	^+^ HA HDEC, ^+^ CRL-2586 OEMA	Cocktail MEHP, DEHP, DCHP and BBP	miR-655 ^(BBP)^ miR-182	Down NE	[44]
Scarano et al., 2019	In vivo Pregnant rat exposure/Ventral prostate tissues from puppies	Cocktail DEHP, DEP, DBP, DiBP, BBzP, DiNP	miR-30d-5p	Up	[41]
			miR-30b-5p	Up	
			miR-141-3p	Up	
			miR-30d-3p	Up	
			mir-184	Up	
Chorley et al., 2020	In vivo Serum and liver tissue of mice	Cocktail DEHP BBP and DNOP	miR-182−5p ^(DEHP)^	Up	[46]
			miR-378a−3p ^(DEHP)^	Up	
			miR-125a−5p	Up	
Zota et al., 2020 (FORGE) study	In vivo Human Fibroid and myometrium tissue—Uterine Leiomyoma	Cocktail ΣDEHP and ΣAA phthalates	miR-10a-5p	Up	[47]
	Myometrium		miR-10a-3p	Up	
	Myometrium		miR-140-3p	Up	
	Myometrium		miR-144-5p	Up	
	Myometrium		miR-150-5p	Up	
	Myometrium		miR-205-5p	Up	
	Myometrium		miR-27a-5p	Up	
	Myometrium		miR-29b-2-5p	Up	
	Myometrium		miR-29c-5p	Up	
	Myometrium		miR-451a	Up	
	Myometrium		miR-95-3p	Up	
	Fibroid		miR-135a-5p	Up	
	Fibroid		miR-135b-5p	Up	
	Fibroid		miR-137-3p	Up	
	Fibroid		miR-302b-3p	Up	
	Fibroid		miR-335-3p	Up	
	Fibroid		miR-34a-5p	Up	
	Fibroid		miR-34a-3p	Up	
	Fibroid		miR-34b-5p	Up	
	Fibroid		miR-34c-5p	Up	
	Fibroid		miR-483-5p	Up	
	Fibroid		miR-488-3p	Up	
	Fibroid		miR-488-5p	Up	
	Fibroid		miR-508-3p	Up	
	Fibroid		miR-577	Up	
	Fibroid		miR-592	Up	
	Fibroid		miR-651-5p	Up	
	Fibroid		miR-885-5p	Up	
	Fibroid		miR-9-3p	Up	

^++^ List of studies are organized according to chronological order in subgroup on the type of plasticizer/cocktail of plasticizers alphabetical basis basis. * AS52-mutant cell (ASMC) clones; ** Human endometrial cancer cell line; ^+^ Human Hemangioma cells; ^β^ Acute myeloid leukemia; ^§^ Human ovarian cancer cell lines; ^^^ Human breast cancer cells; ^ρ^ (ERα-negative and estrogen-resistant); NE: no effect; UP: upregulated, Down: downregulated, ° Human hepatocellular carcinoma; ^Σ^ Human oral squamous cell carcinoma; ^π^ Human prostate cancer cells; ^$^ cancer cells; ^a^ (10^−4^ or 10^−5^ M); ^b^ (10^−6^ to 10^−11^ M); Mono-ethylhexyl phthalate (MEHP); Bis (2-ethylhexyl) phthalate (DEHP); diethyl-phthalate (DEP); dibutyl phthalate (DBP); di-isobutyl-phthalate (DiBP), butylbenzyl-phthalate (BBzP); di-isononyl-phthalate (DiNP); benzyl butyl phthalate (BBP); 4-nonylphenol (NP); 4-tert-octylphenol (OP); di-noctyl phthalate (DNOP); Tris (1,3-dichloro-2-propyl) phosphate (TDCIPP) = organophosphate flame retardants. Observational Research on Genes and the Environment (FORGE) study; ΣDEHP = Sum of 21 phthalates and metabolites; ΣAA phthalates = Sum of 31 antiandrogenic phthalate metabolites.
